# Characterization of airborne dust samples collected from core areas of Kathmandu Valley

**DOI:** 10.1016/j.heliyon.2020.e03791

**Published:** 2020-04-24

**Authors:** Bhanu B. Neupane, Amita Sharma, Basant Giri, Mahesh K. Joshi

**Affiliations:** a Central Department of Chemistry, Tribhuvan University, Kirtipur, Kathmandu, Nepal; b Center for Analytical Sciences, Kathmandu Institute of Applied Sciences, Kathmandu, Nepal; c Department of Chemistry, Tri-chandra Campus, Tribhuvan University, Kathmandu, Nepal

**Keywords:** Particulate matter, Clay minerals, Asbestos, Cement, Microscopy materials science, Chemistry, Environmental science, Earth sciences, Health sciences

## Abstract

Kathmandu Valley is reported to be one of the highly polluted and populated cities in the world. Particulate matter is one of the major contributors of unhealthy air in Kathmandu. Although there are several reports on spatial and temporal variation of air quality of Kathmandu Valley, the morphological and mineralogical characteristics of particulate matter are very limited or none. In this study, we report on the mineralogical and morphological analysis of airborne particulate matter collected from densely populated core areas of Kathmandu Valley using spectroscopic and microscopic techniques. The Fourier Transform Infrared (FTIR) and X-ray Diffraction (XRD) data showed the presence of clay minerals, crystalline silicate mineral, carbonate minerals, and asbestiform mineral in the dust samples. The field emission scanning electron microscopic analysis confirmed the existence of particles having diverse morphology with some of the particles having aspect ratio as high as twenty; indicating the existence of asbestiform type minerals. Based on SEM-EDX data, we found that the relative distribution of elements to be different in different samples and C, O, Mg, Ca, and Si were the major elements in the dust samples. Interestingly, the XRD data analysis showed that in all the samples quartz mineral having high degree of crystallinity was present. The XRD measurement was also carried out in three different brands of cement samples. Few minerals present in dust samples were also identified in the cement samples. This observation could indicate that cement is one of the sources of minerals in the airborne particulate matter in the Kathmandu Valley.

## 1 Introduction

Air pollution is one of the major threats to climate, human health, and ecosystems and is one of the highly discussed global issues [1, 2, 3, 4]. It is reported that the combined effect of outdoor and indoor air pollution accounts for around 7 million global deaths annually [5]. According to the most recent air quality database, around 97% of the cities in the low- and middle-income countries and 49% of cities in high income countries having population more than 100,000 do not meet the world health organization (WHO) air quality guideline [5].

The airborne particles of size 10 μm or less (PM_10_) and 2.5 μm or less (PM_2.5_) are one of the major air pollutants that can penetrate deeply into the respiratory system. Exposure to these particles is linked to different health issues, such as chronic bronchitis, asthma, diabetes, stroke, cancer and eventually death [5, 6, 7]. The global burden of disease (GBD) methodology estimated that ambient PM_2.5_ pollution is the fifth leading cause of death causing 4.2 million deaths and 103.1 million disability adjusted life years in 2015 [8].

Health effect of particulate matter depends on multiple parameters such as exposure dose, chemical composition, morphological characteristics, surface reactivity, hydrophobicity and hydrophilicity, and solubility after deposition in the targeted sites [9, 10, 11, 12, 13]. Inhalation of asbestiform particulate matter, depending on the nature and exposure dose, is reported to cause pleural fibrosis, asbestosis, carcinoma of lung, and mesothelioma [10]. Similarly, inhalation of crystalline silica (quartz) is reported to tuberculosis, silicosis, chronic bronchitis/chronic obstructive pulmonary disease (COPD) and lung cancer while amorphous silica is found to be very less toxic [14, 15]. Airborne particulate matter also contains clay minerals of different types. The inhalation of clay mineral rich particulate matter in occupational place is, however, reported to have minimal or no toxic effects [16]. Airborne particulate matter having high content of heavy metals, such as lead, arsenic, and mercury is reported to cause health effects ranging from neurotoxicity to cancer [4]. Besides human health effects, impact of airborne mineral particles on the climate and ecosystems is also an active area of research [12, 17, 18].

Kathmandu Valley, the largest and capitol city of Nepal, has witnessed increase in the particulate matter pollution in recent decades. The PM_2.5_ in Kathmandu has been reported to be exceeding the WHO guideline for most of the months [19, 20]. There are few studies that correlated the particulate matter exposure to various health issues in Kathmandu especially the COPD and cardiovascular diseases [21, 22, 23, 24]. The major source of air pollution in Kathmandu has been attributed to vehicular emission, brick kiln emission, emission from waste burning, and dust from agricultural soil and construction material [21, 25, 26].

The chemical composition of fine particulate matter of Kathmandu Valley (KV) are also reported recently. It was reported that concentration of organic carbon, inorganic ions, and heavy metals in the PM_2.5_ collected form near roadside areas was seasonal dependent. Calcium, aluminum, silica, calcium, and iron were the most abundant elements during both monsoon and spring seasons [27]. A study on molecular characterization of organic aerosol and their primary and secondary sources in suburban site of Kathmandu from April 2013 to April 2014 was made by Wan et al. and reported that the concentrations of organic carbon (OC) and elemental carbon (EC) increased during winter with a maximum monthly average observed in January [28]. The organic carbon was found to be mainly originated from biomass burning and other anthropogenic activities in Kathmandu.

Several studies have suggested that mineral distribution and composition of airborne particulate matter is important to understand the health impact of a region [13, 29, 30, 31, 32, 33, 34, 35]. Recently, we reported an optical microscopic study on the air dust samples collected from different core areas of Kathmandu Valley in April 2018 [36]. It was found that significant number particles in the samples to be asymmetric. However, a systematic study on the mineral content, elemental analysis and morphology of air dust samples of Kathmandu is not reported in literature yet. Construction and demolition works are happening all the times in Kathmandu. Standard protocol is often not followed in transportation, construction, and demolition of the materials and structures. These activities can result in generation of mineral rich particulate matter.

In this research, we made mineralogical and morphological analysis of the airborne dust samples collected from eleven densely populated locations of Kathmandu Valley. We used FTIR and XRD measurement to identify the minerals. The information on the morphology and elemental composition of sample was obtained from SEM and SEM-EDX, respectively. We also analyzed XRD data of all the samples to find out the crystallinity index of silicate mineral. The XRD measurements were also made in three different brands of cement samples.

## 2 Materials and methods

### 2.1 Sample collection

**Figure 1:**
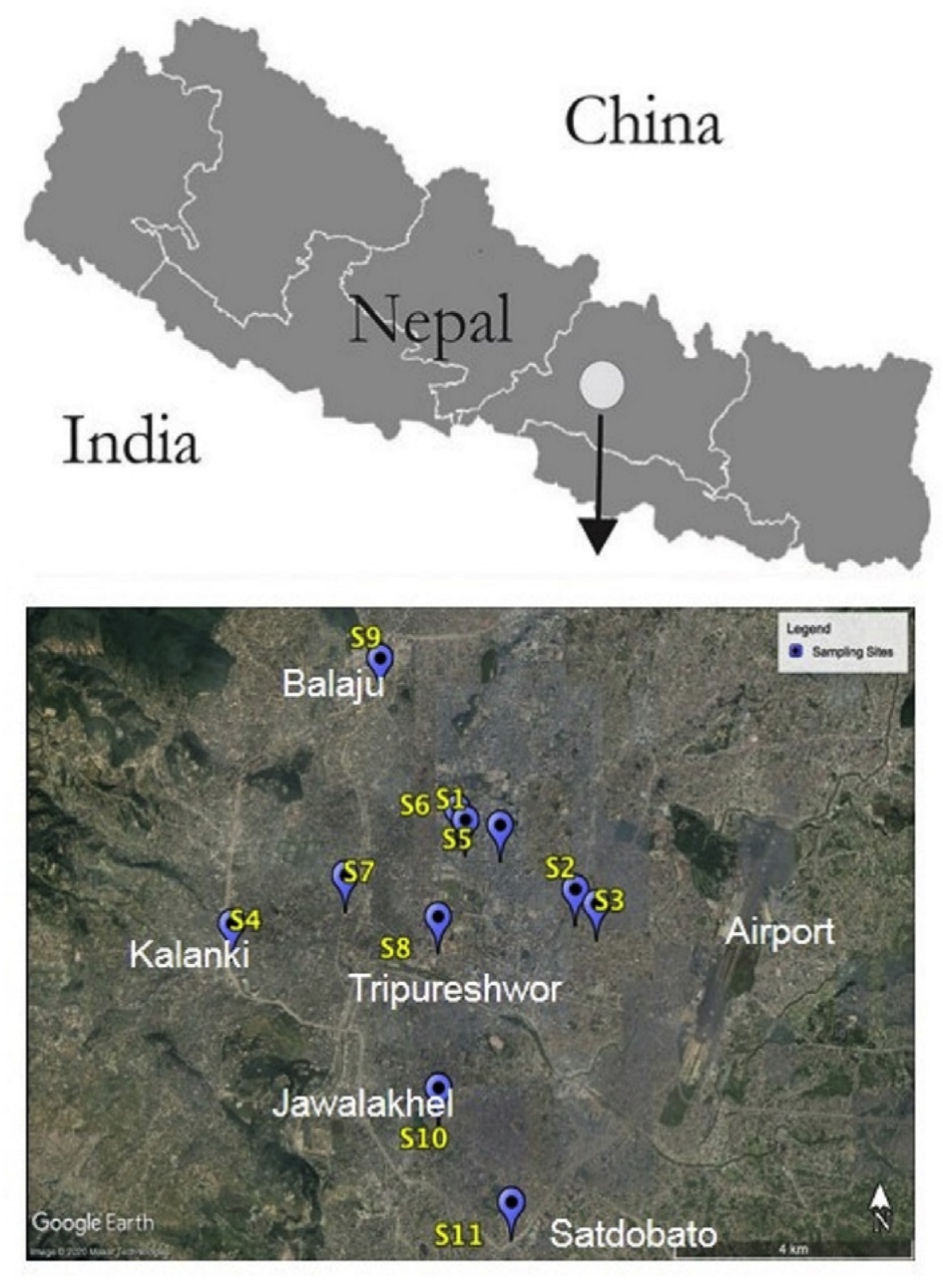
A part of Google Earth map showing sampling sites S1 to S11.

The passive sampling method was adopted in this study. The free-falling particles were collected in a sterile polystyrene petri dish (100 mm internal diameter and 15 mm depth, Fisher Scientific) in dry sunny days in March 2018. The petri dishes were placed at a height of 10 m above the ground in an open box. The samples were collected from eleven densely populated core locations of Kathmandu Valley. The samples were collected over a period of 24 h at each location. The sample collection locations are shown in Figure 1. The detail information on the sampling sites is provided in Table 1.

**Table 1:** Details on sampling sites.

Sample ID	Location name	Location coordinates
S1	Bagbazar	27°42′22.93″N, 85°19′3.93″E
S2	Old Baneshwor	27°41′51.29″N, 85°20′3.57″E
S3	Ratna Rajya School, Baneshwor	27°41′44.55″N, 85°20′14.74″E
S4	Kalanki	27°41′35.49″N, 85°16′57.58″E
S5	Putalisadak	27°42′20.52″N, 85°19′22.73″E
S6	Trichandra Campus, Ghantaghar	27°42′28.21″N, 85°18′59.46″E
S7	Kalimati	27°41′57.57″N, 85°17′58.59″E
S8	Tripureshwor	27°41′38.81″N, 85°18′49.12″E
S9	Balaju	27°43′38.64″N, 85°18′16.40″E
S10	Jawalakhel	27°40′22.18″N, 85°18′49.62″E
S11	Satdobato	27°39′32.48″N, 85°19′28.19″E

### 2.2 Fourier transform infrared study

The infrared spectra of all the samples were collected in the range of 4000–400 cm^−1^ by a Fourier transform infrared spectrometer (ABB Bomen MB100, Canada) in an attenuated total reflection mode (ATR). Before measuring the FTIR spectra of sample, background spectrum of ATR crystal was measured and subtracted from the sample spectrum. The spectral resolution of the spectrometer was 4 cm^−1^. Each reported spectrum is the average of sixteen scans that provided excellent signal to noise ratio. The different minerals in the dust sample was identified on the basis of literature studies and RRUFF database of reference standard [37].

### 2.3 Electron microscopic study

The electron microscopic images and energy dispersive X-ray (EDX) spectra were measured by a field emission scanning electron microscope (FE-SEM, Carl Zeiss, Supra 40VP, Japan). For SEM measurement, the sample was mounted on aluminum stub using double sided carbon tape. To make the sample surface conductive, a very thin layer of platinum was coated on the sample using a sputter coater. The sample was then placed in the vacuum chamber of SEM instrument and image was collected. For each sample 5–10 SEM images were taken at different magnifications. Image analysis was done in ImageJ software; a Java-based open source developed by National Institutes of Health and the Laboratory, USA. Aspect ratio of a particle was calculated by dividing the highest dimension (length) of the particle by its lowest dimension (width).

The SEM-EDX consisted of a silicon drift detector having detection limit of around 0.1% and capable of collecting spectrum from specific points and field of view for qualitative and semi-quantitative elemental analysis. In EDX measurement, we measured full field of view EDX spectra of all the samples. We also measured single particle spectra in few samples. The EDX energy window was calibrated following manufacturer's specification and the calibration was checked for the peak accuracy using standard lines/peaks. The ZAF correction was applied to EDX data to get semi-quantitative information. Here, Z refers to the atomic power correction that considers the stopping power of an element, A the absorption correction, and F is the fluorescence correction.

### 2.4 X-ray diffraction measurement and analysis

The X-ray diffraction data were collected at Bragg angle 2θ ranging from 10 to 80° (step size 0.01^o^) by X-ray diffractometer (Rigaku, UK). The Cu Kα line having wavelength of 1.540 A° (30 kV, 40 mA) was used as X-ray source. The inter-planar spacing (d_hkl_) was calculated by using the Braggs' equation nλ = 2d_hkl_Sinθ, where n is order of reflection (n = 1) and λ is wavelength of X-ray used (λ = 1.54Å). The parameters such as peak positions (2θ) and inter-planer spacing (d_hkl_) were compared with the literature data and RRUFF database of reference standard to identify the different minerals present in the samples [37].

The crystallinity index (CI) of the quartz mineral in the samples was measured in the scale of 0–10 by using the formula CI = 10 *aF/b*; where *F* is experimental parameter (which is ~1.65), *b* is total intensity of peak at 67.8° (including amorphous background), and *a* is intensity of crystalline peak only [38].

## 3 Results

### 3.1 SEM and SEM-EDX measurement in the dust samples

**Figure 2:**
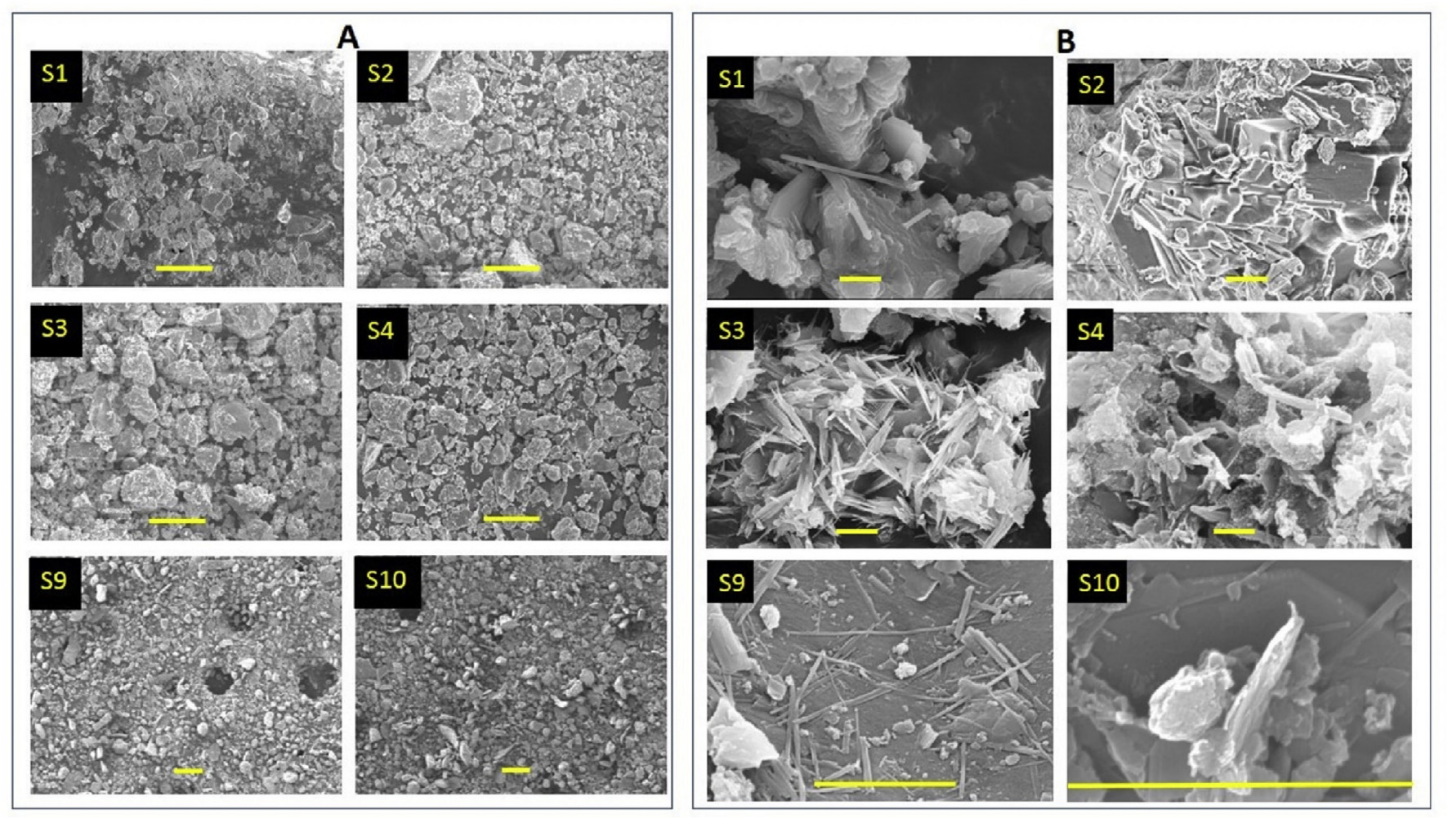
FE-SEM images of selected samples. Scale bar in frame A and B is 100 and 1 μm, respectively.

We measured the FE-SEM images of all the samples (S1–S11) at different magnification. Representative images for the samples S1, S2, S3, S4, S9, and S10 are shown in Figure 2A and B.

**Figure 3:**
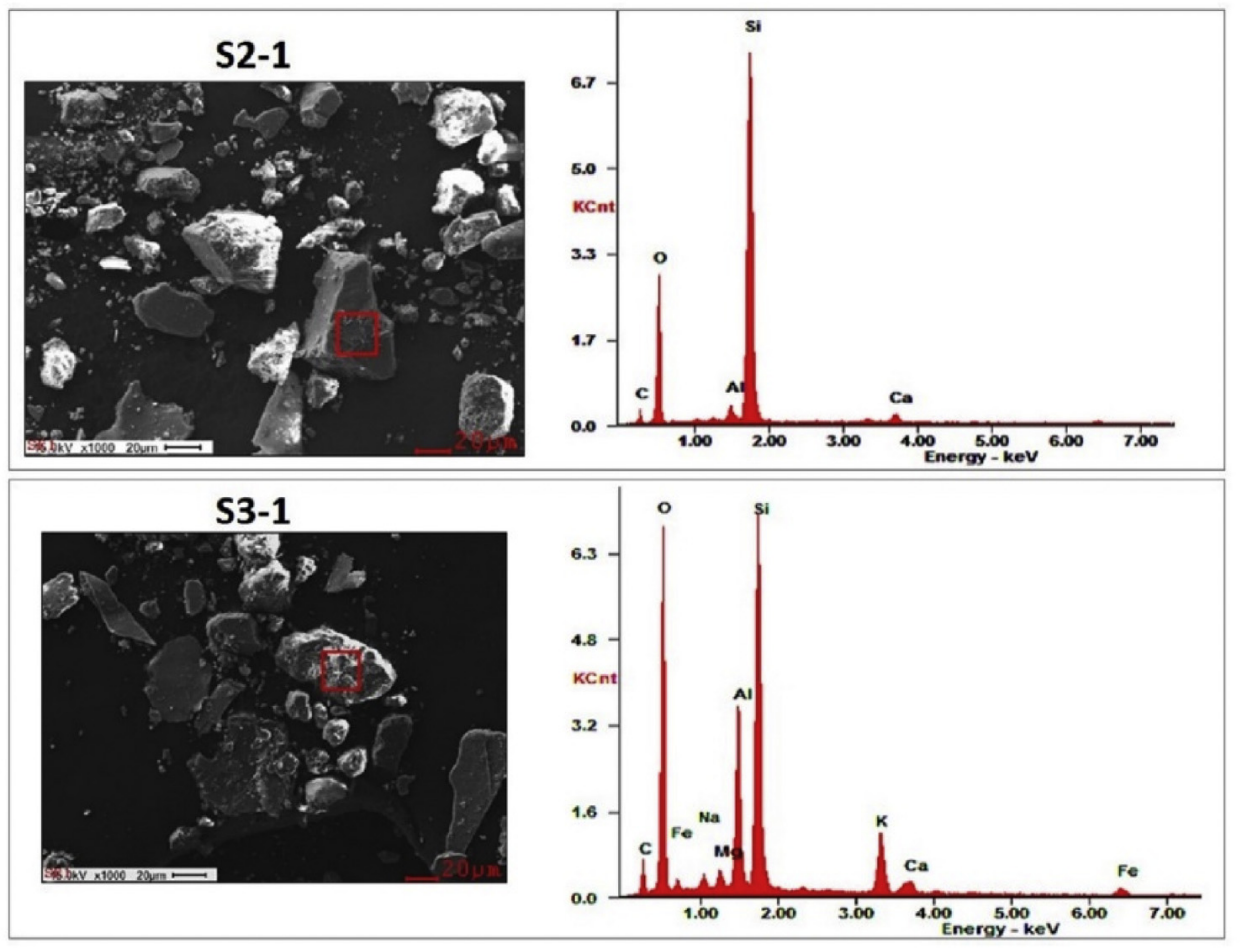
The SEM-EDX spectra of the selected regions (as marked by red square box in left frames) in the samples S2 (S2-1) and S3 (S3-1).

We measured EDX data in samples S1–S11 and also the single particle EDX in the few selective particles/regions of the samples S2 and S3 to understand the elemental composition of the dust samples. The SEM image and the single particle EDX spectra for sample S2 and S3 are shown in Figure 3.

The atomic weight percentage distribution obtained from the EDX spectrum of all the samples is provided in Table 2.

**Table 2:** EDX and single particle EDX data of different samples.

Samples	Atomic weight percentage of different elements												
	C	O	Na	S	Cl	Mg	Al	Si	K	Ca	Fe	Mo	Ni
S1	10.56	56.49	-	-	-	0.17	0.64	9.24	0.38	22.51	-	-	-
S2	9.26	61.98	0.19	-	-	0.61	8.14	10.59	3.74	3.93	1.36	-	0.19
S2-1	14.88	44.42	-	-	-	-	1.55	37.85	-	1.30	-	-	-
S3	10.23	55.56	0.18	-	-	0.27	1.19	9.23	0.45	23.16	0.18	-	-
S3-1	14.19	50.66	0.77	-	-	0.86	8.50	19.13	3.74	0.84	1.31	-	-
S3-2	6.11	51.27	0.86	-	-	0.95	13.69	20.15	4.36	0.56	1.63	-	-
S4	14.40	52.5	1.0	-	-	1.3	6.73	1.56	1.30	9.99	1.24	0.86	-
S5	14.44	30.52	0.27	3.55	1.92	1.22	8.69	18.71	5.23	15.31	0.12	-	-
S6	47.33	34.67	0.80	-	-	0.60	3.66	10.33	1.10	0.36	1.24	-	-
S7	21.50	44.01	-	-	-	0.53	1.74	4.58	0.69	25.56	0.34	-	-
S8	11.41	59.81	0.21	-	-	6.01	9.13	9.01	0.84	0.43	3.58	-	-
S9	26.13	43.77	0.73	0.24	-	0.66	5.76	17.01	2.10	2.35	1.24	-	-
S10	14.57	49.69	4.04	-	-	0.76	8.79	19.39	1.04	0.72	0.99	-	-
S11	12.28	52.19	0.65	-	-	3.04	10.37	15.63	2.12	2.38	1.32	-	-

### 3.2 FTIR measurement in the dust samples

**Figure 4:**
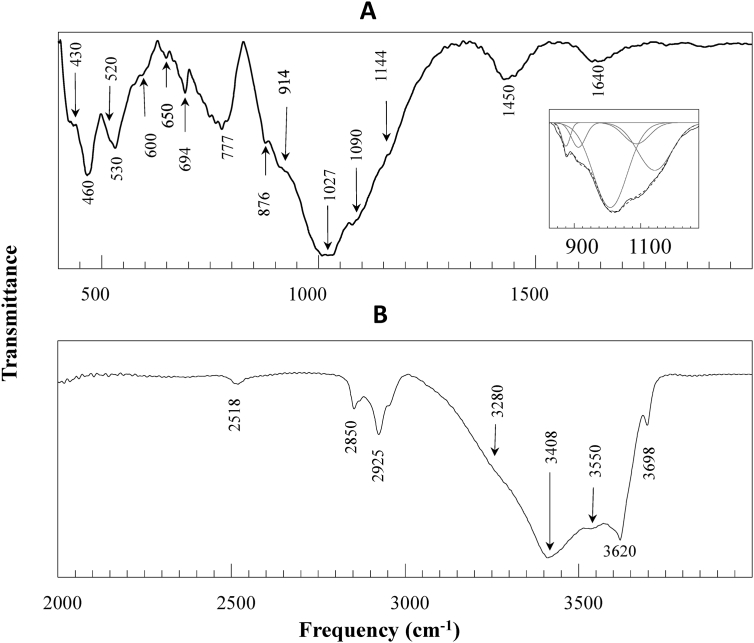
FTIR spectrum of sample 11 (S11). For clarity, the spectrum collected in the middle IR (MIR) range (400-4000 cm^−1^) is plotted in two separate windows 400–1999 (frame A) and 2000-4000 cm^−1^ (frame B). The inset in A shows a five component Gaussian fit of the spectrum in the range of 830–1250 cm^−1^.

We measured FTIR spectra of all the samples (S1 to S11) in attenuated total reflectance (ATR) mode. The FTIR spectrum of sample S11 is shown in Figure 4. For clarity, the spectrum collected in the middle IR (MIR) range is plotted in two separate frames, 400-1999 cm^−1^ in frame A and 2000-4000 cm^−1^ in frame B. To get more accurate peak/shoulder position in the range of 830–1250 cm^−1^, a five component Gaussian fitting was used and the result is shown as inset in frame A.

**Figure 5:**
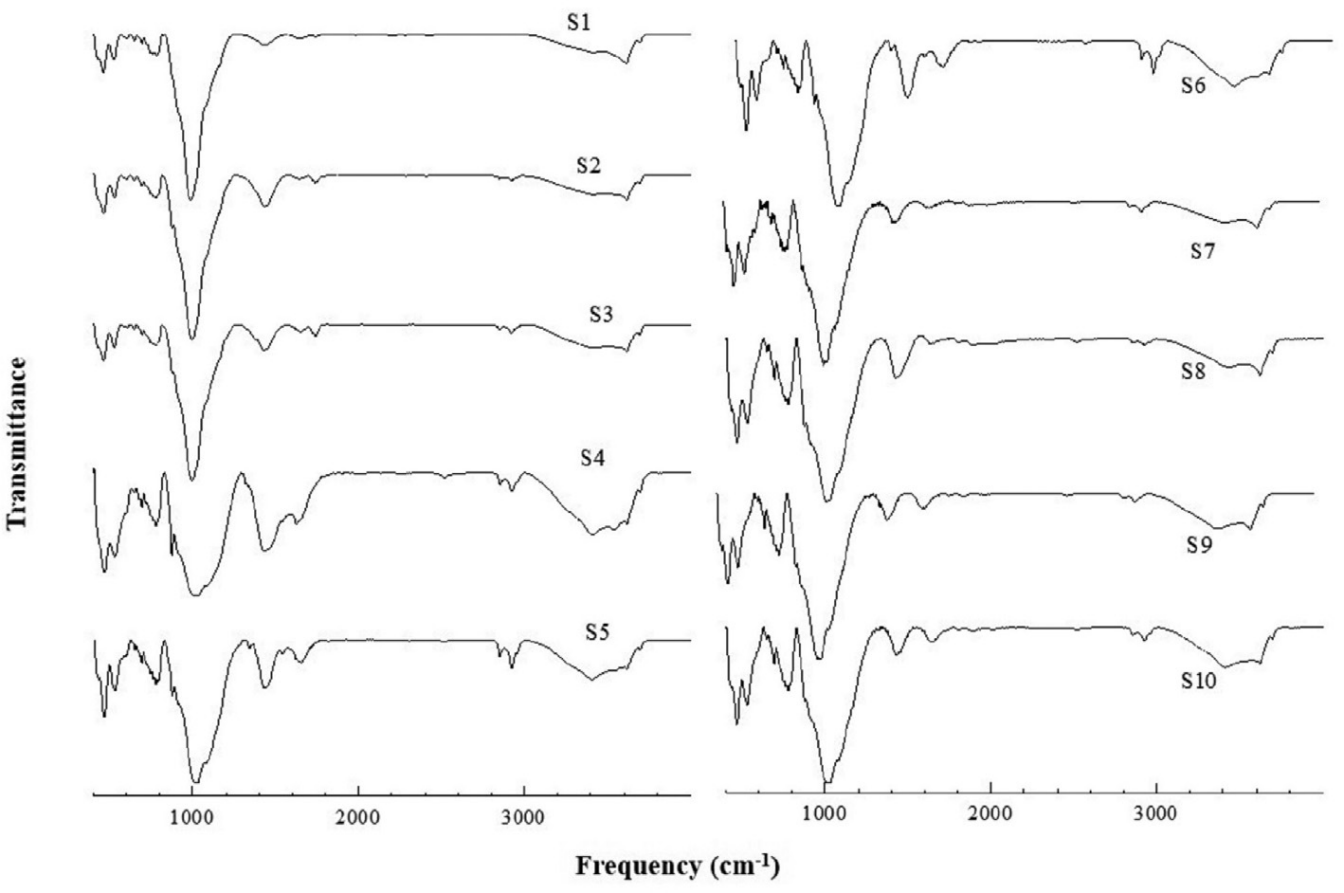
FTIR spectra of samples S1–S10.

The FTIR spectra of the remaining samples (S1–S10) in the range 400–4000 cm^−1^ is provided in Figure 5.

### 3.3 XRD measurement

**Figure 6:**
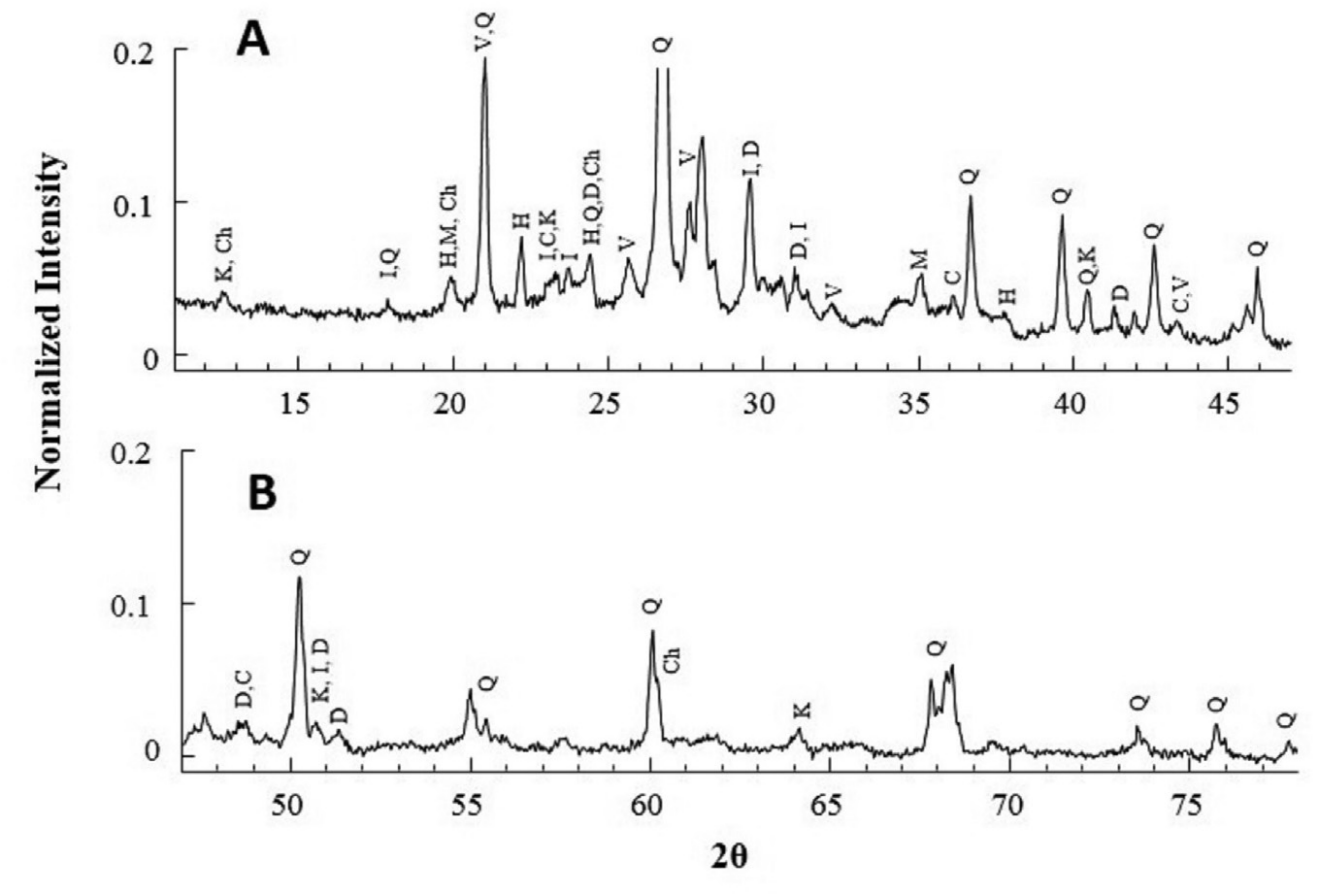
The XRD datum for S11. The peaks corresponding to different minerals are indicated by different letters. D = dolomite, M = montmorrilonite, C = calcite, Q = quartz, K = kaolinite, I = illite, H = hematite, V = vaterite, and Ch = chrysotile.

We measured the X-ray diffraction data of the dust samples and found that the XRD data for all samples were similar. The XRD datum of S11 is shown in Figure 6. For clarity, a separate plot for 2θ value in the range 10–46.5^o^ and 46.5–78^o^ is made in frames A and B, respectively. For easy comparison, intensity is normalized at the most intense peak (2θ = 26.6^o^) and the peak height is truncated.

Minerals in the dust samples were identified by comparing the characteristic X-ray diffraction peaks to the literature data and an open source RRUFF database of reference standards [37]. We refer to section 4.3 for details on the assignment XRD peak and identification of minerals. In Figure 6 the letters D is for mineral dolomite, C for calcite, Q for quartz, K for kaolinite, I for illite, H for hematite, V for vaterite, and Ch for chrysotile.

**Figure 7:**
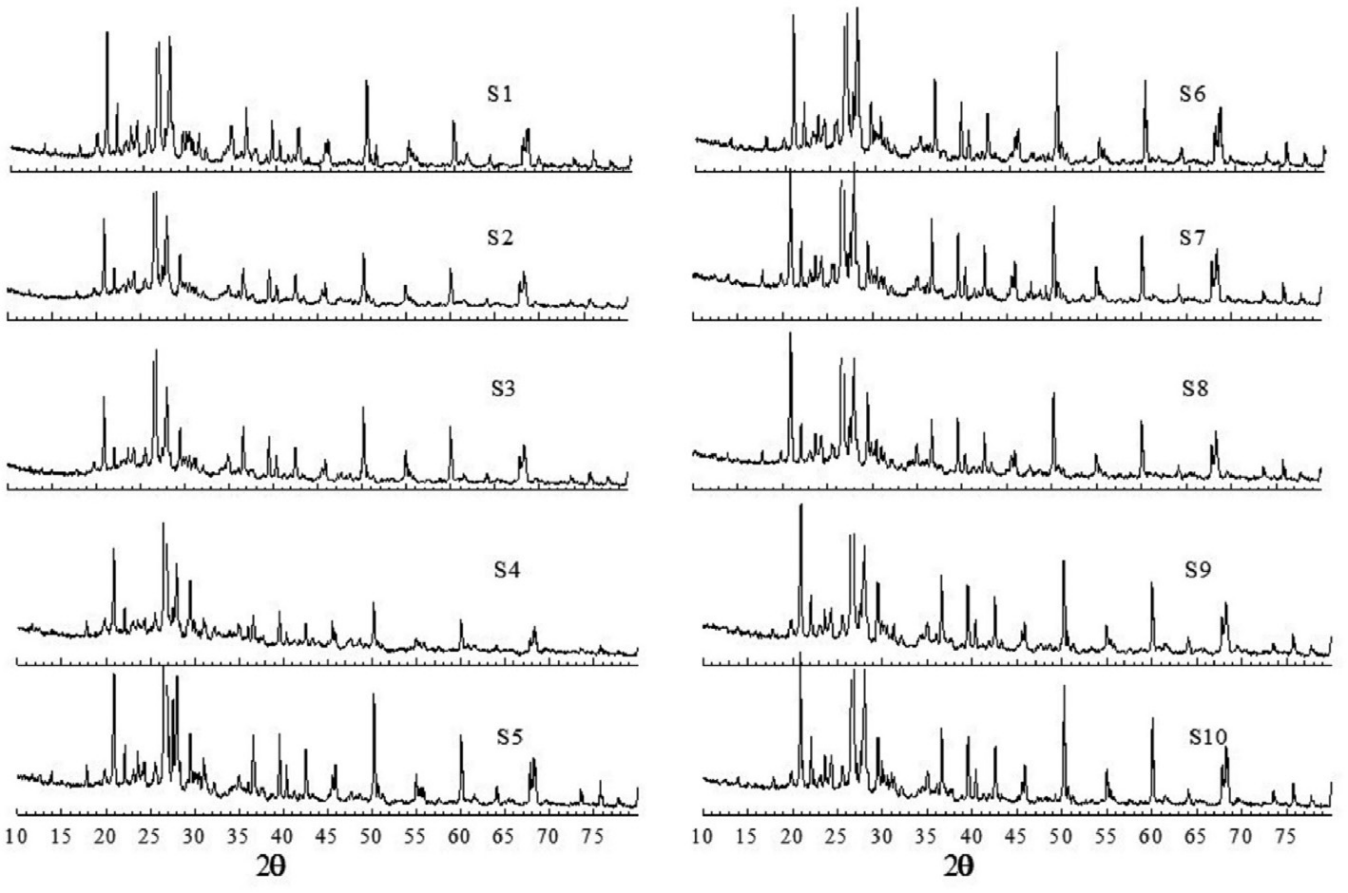
XRD data of the samples S1–S10.

The XRD data of the samples S1–S10 is shown in Figure 7.

## 4 Discussion

### 4.1 FE-SEM and EDX measurements

The FE-SEM images indicated that most of the dust particles were symmetrical (lower aspect ratio in the range of 1–3) (Figure 2A) with few particles with diverse morphology (Figure 2B) and aspect ratio greater than three. For example, sample S3 consisted of sheet like particles having multiple micro fibrils and sample S9 ribbon shaped particles having aspect ratio as high as twenty. Such particles can be classified as asbestiform particles. This observation is in consistent to the recent study where particles of diverse morphology having aspect ratio as high as 20 was reported in dust samples of Kathmandu Valley [36].

On the basis of data in Table 2, we found that the dust samples contain metallic elements Fe, Mg, Al, Si, Ca, and Na, and non-metallic elements C and O distributed in different ratio. Calcium and silicon were the most abundant elements.

### 4.2 FTIR measurement in the dust samples

The Fourier transform infrared spectroscopy (FTIR) is one of the highly used techniques to characterize minerals present in dust samples [33, 39, 40, 41, 42]. The Si–O bending vibration modes at 465 cm^−1^ and 694 cm^−1^ and stretching modes at 777 cm^−1^, 800 cm^−1^, 1090, 1144 cm^−1^, and 1872 cm^−1^ are the characteristic frequencies of quartz [33, 40, 41, 42, 43, 44, 45]. These frequencies were observed in all the samples reported in this study (Figures 4 and 5). Quartz is one of the most abundant crystalline silicate minerals that contains a continuous framework of SiO_4_ tetrahedra. The epidemiological studies have reported that inhalation of crystalline silica dust, depending on the exposure dose, can lead to inflammation, pulmonary tuberculosis, silicosis, and lung cancer [31, 46].

Clay minerals such as montmorillonite, kaolinite, and illite were reported in dust samples collected from different cities of the world. The absorption peaks near 3620 cm^−1^ and 3550 cm^−1^ were assigned to stretching frequency of O–H bond of H_2_O bound directly to interlayer cations (inner sphere) and O–H of surface bonded water in montmorrilonite [39]. The characteristic bending mode of AlAlO–H group in montmorrilonite was reported to exist at 914 cm^−1^. These characteristic frequencies were found in S1, S2, S3, S4, S5, S6, and S11 (Figures 4 and 5), thus indicating the presence of montmorrilonite in these samples. Montmorrilonite is a pyllosilicate group of clay minerals (layered alumino-silicate) that can form microscopic crystals called clay. Chemically it is hydrated sodium calcium aluminum magnesium silicate hydroxide (Na,Ca)_0.33_(Al, Mg)_2_(Si_4_O_10_) (OH)_2_·*n*H_2_O and ions such as potassium and iron can exist as substituents. Montmorrilonite forms layered structure. A number of cations and water molecules can exist in the interlayer region thereby making a mineral having high shrink-swell and cation exchange capacity.

The absorption peak at 3698 cm^−1^ was assigned to stretching frequency of structural O–H, of Kaolinite [47]. It was suggested that the peak helps to identify kaolinite from other clay minerals without the use of other complimentary techniques. We found this feature in all the samples and along with other frequencies (see Table 3) confirm the presence of kaolinite in the samples. Kaolinite is a clay mineral having low shrink-swell and cation exchange capacity. It is represented by molecular formula Al_2_Si_2_O_5_(OH)_4_. It is a layered silicate mineral in which tetrahedral sheets of silica (SiO_4_) are linked through oxygen atoms to the octahedral sheet of alumina (AlO_6_).

**Table 3:** The vibrational frequencies observed in FTIR spectra of different samples (S1–S11) and assignment to the corresponding minerals.

Frequency (cm^−1^)	Nature of band	Assignment
3698	sharp peak	stretching of structural O–H of kaolinite
3688, 3691	shoulder	stretching of structural (surface) O–H of chrysotile
3645	shoulder	average envelope of stretching of structural (inner) O–H of chrysotile and O–H of crocidolite
3620	sharp peak	average envelope of O–H stretching of intercalated water of kaolinite, stretching of structural O–H of crocidolite, montmorillonite and illite, stretching of O–H of water bounded directly to cation (Fe^+++^ or Ca^++^) in the interlayer region of montmorillonite
3550	broad peak	O–H of surface bonded water in montmorrilonite
3405	broad peak	O–H stretching of surface adsorbed water in clay minerals
3280	shoulder	bending overtone of surface adsorbed water
2520	broad peak	combination mode of calcite and vaterite
2925	sharp peak	C–H stretching (hydrocarbon)
2850	sharp peak	C–H stretching (hydrocarbon)
1805	weak peak	combination mode of calcite and vaterite
1740	broad peak	C=O stretching (organic compounds)
1640	broad peak	O–H bending (surface water)
1450	broad peak	stretching mode carbonate C–O (calcite, vaterite)
1143	shoulder	Si–O stretching (quartz)
1087	shoulder	average envelope of Si–O stretching of quartz and combination mode of calcite and vaterite
1027∗	broad peak	average envelope of Si–O stretching of montmorillonite, kaolinite, illite, crocidolite, chrysotile
914	weak shoulder	deformation/bending vibration of structural OH of kaolinite, montmorillonite, illite, chrysotile
876	weak peak	out of plane bending of carbonate C–O of calcite and vaterite
882	weak shoulder	out of plane bending of carbonate C–O of dolomite
777	broad peak	silicate chain vibration of quartz and crocidolite
750	weak peak	Al–O–Si in plane vibration of illite,
745	weak peak	in plane bending of carbonate C–O of vaterite
730	weak shoulder	Al–Mg–OH deformation of illite, in plane bending of carbonate C–O of dolomite
715	weak peak	in plane bending of carbonate C–O of calcite
694	weak peak	silicate ring vibration of quartz, kaolinite, and crocidolite
670	weak peak	silicate ring vibration crocidolite
652	broad weak peak	average envelope of silicate ring vibration of crocidolite and vibration of outer Mg–OH of chrysotile
605	weak shoulder	vibration of inner Mg–OH of chrysotile
541, 505	weak shoulder	cation oxygen stretching of crocidolite
530	broad peak	Fe–O stretching of hematite
524	weak shoulder	Al–O–Si deformation montmorillolinte and illite
471	weak shoulder	Si–O–Si deformation of illite and chrysotile, bending vibration of Mg–O bond of chrysotile
452	weak peak	Fe–O bending of hematite
460	broad peak	average envelope of Si–O–Si deformation of quartz and montmorillonite
436	broad peak	Si–O–Si deformation of illite and chrysotile, bending vibration of Mg–O bond of chrysotile
425	weak shoulder	Si–O–Si deformation of kaolinite

∗spectral de-convolution shows this peak around 1010 cm^−1^.

Illite is difficult to confirm in presence of other clay minerals. However, the presence of structural O–H stretching around 3620 cm^−1^ coupled with Al–Mg–OH deformation at around 830 cm^−1^ and Al–O–Si in plane vibration around 750 cm^−1^ may indicate illite [48]. These characteristic peaks along with other frequencies (given in Table 3) were present in all samples indicating illite. Illite is also a clay mineral that falls under the pyllosilicate group of minerals (K,H_3_O) (Al,Mg,Fe)_2_(Si,Al)_4_O_10_[(OH)_2_(H_2_O)]. It is non-expanding clay mineral having cation exchange capacity similar to kaolinite. Illite consists of sandwich structure of silica tetrahedron–alumina octahedron–silica tetrahedron layers.

Different carbonate minerals, such as polymorphs of CaCO_3_ (calcite, vaterite, aragonite), dolomite, thermonatrite are some of the commonly reported carbonate minerals in atmospheric dust samples [29, 30, 31, 32, 49]. Vaterite is one of the highly abundant polymorph of CaCO_3_. It can be identified in FTIR spectum by the existence of out-of-plane bending mode near 876 cm^−1^ (ν_2_), in-plane bending mode near 745 cm^−1^(ν_4_), stretching modes near 1087 cm^−1^ (ν_1_) and 1430 cm^−1^ (ν_3_), combined modes 1800 cm^−1^(ν_1_+ ν_4_) and 2520 cm^−1^ (ν_1_+ ν_3_) [49–51]. The vibrational frequencies characteristic of vaterite were present in all samples.

Calcite is the next most abundant polymorph of CaCO_3_. It can be identified by the presence of in-plane bending mode near 715 cm^−1^ (other modes being same as of vaterite) [49, 50, 51]. The vibrational frequencies characteristic of vaterite were present in all samples. Dolomite can be identified in IR spectra by the existence of ν_4_ mode at 730 cm^−1^ ν_2_ mode at 882 cm^−1^, but the ν_1_, ν_1_+ ν_4_, ν_1_+ ν_3_ modes overlap to that of calcite and vaterite [51]. These vibrational frequencies characteristic of dolomite were present in all samples. Dolomite is anhydrous mixed carbonate of calcium and magnesium and is represented by CaCO_3_. MgCO_3_.

Iron rich minerals are also reported in dust samples and hematite is one of the most abundant iron minerals having formula of Fe_2_O_3_. This mineral can be identified by the Fe–O stretching frequency at 530 cm^−1^ and bending frequency at 452 cm^−1^ [52]. These frequencies were present in all samples indicating the presence of hematite in all samples.

Presence of asbestiform minerals in atmospheric dust samples was reported in several studies [29, 30, 33, 53]. Asbestos is the generic term used for naturally occurring crystalline silicate minerals having thin and long fiber with each fiber composed of many micofibrils. According to the physical make-up of the fiber, asbestiform minerals can be classified into serpentine and amphiboles groups. The serpentine group is characterized by curly fibers. Chrysotile (also known as white asbestos) falls in this group. The amphibole group of minerals contain pointed fibers and anthophyllite, crocidolite (also known as blue asbestos), actinolite, amosite (also known as brown asbestos), and tremolite fall in this group [54, 55]. It is well documented that inhalation of dust containing asbestiform minerals, depending on the expose dose and type, cause multitude of health issues, such as pleura, asbestosis, lung cancer, mesothelioma and other cancers [10, 56, 57, 58].

FTIR measurement in our sample showed vibrational frequencies of two asbestiform minerals chrysotile and crocidolite. Depending on the origin, it is reported that the stretching frequency of (structural) surface O–H of chrysotile appears in the range of 3688–3697 cm^−1^ and stretching of inner O–H appears in the range of 3640–3646 cm^−1^ [59, 60, 61]. In addition, outer Mg–OH vibration frequency at 650 cm^−1^, inner Mg–OH vibration at 604-611 cm^−1^, are the other important frequencies that helps to identify chrysotile form other minerals [59, 60, 61]. These characteristics frequencies in FTIR measurements were present in all dust samples thereby indicating the presence of the asbestiform mineral chrysotile. Chrysotile is a fibrous, soft and disordered asbestos of serpentine group and commonly represented by a chemical formula of Mg_3_(Si_2_O_5_)(OH)_4_. It is one of the most commonly used asbestos and its prolonged exposure is reported to cause lung cancer.

Crocidolite, on the other hand, can be identified by the presence of stretching of the structural O–H at 3645 and 3620 cm^−1^, Si–O stretching vibration at in the range of 990–1143 cm^−1^, silica chain and ring vibration at 778, 725, 694 cm^−1^, and cation-oxygen stretching vibration at 541 and 505 cm^−1^ [59]. These characteristic frequencies were present in samples S6–S11. Crocidolite is reported to be the most toxic form of asbestos. It is the fibrous form of the amphibole mineral riebeckite with chemical formula of Na_2_(Fe^2+^_3_Fe^3+^_2_)Si_8_O_22_(OH)_2_.

The organic carbon can be identified from the presence of C–H stretching frequency at 2925 and 2850 cm^−1^. The organic carbon was detected all the samples. This is expected observation as the sampling was done in the densely populated and vehicular area of Kathmandu.

A summary of the vibrational frequencies observed in FTIR spectra of different samples and their assignment to the corresponding minerals is shown in Table 3.

### 4.3 XRD measurement

In mineralogical study the XRD data are used as the best complementary data to the FTIR data to identify the minerals present in complicated samples, such as atmospheric dust and soil samples. It was reported that the XRD peaks of asbestiform mineral chrysotile appear at 2θ values of 12.1°, 19.6°, 24.4°, 36.8°, 60.1° [60, 62] and crocidolite at 10.5° and 28.8° [63]. The characteristic peaks for chrysotile were found in all samples except S5 and S9 (Figures 6 and 7). Unlike FTIR data discussed earlier, none of the samples shows characteristics peaks that of crocidolite. In XRD spectrum, a perfectly crystalline sample shows more prominent peaks than a less crystalline sample, so all the minerals detected in FTIR may not be detected in XRD due to difference in crystalline properties. This observation is in consistent with literature study where asbestiform minerals were detected in FTIR but not in XRD [33].

The observed XRD peaks (2θ values), calculated inter-planar spacing (d_hkl_) and their assignment for the samples studied in this work shown in Table 4. The XRD pattern characteristic of vaterite [64], calcite [65], dolomite [37], kaolinite [47, 65, 66], quartz [67], illite [68], hematite [69] were present in all samples thereby confirming the presence of these minerals in all samples. The conclusion made here is consistent with FTIR analysis made in earlier study.

**Table 4:** The peak position, inter-planar spacing, and their assignment for different minerals.

Minerals	Samples	2θ (°)	dhkl (Å)
Illite	S1–S11	17.8, 23.7, 23.08, 26.3, 28.90, 31.3, 45.90, 50.36	4.99, 3.76, 3.86, 3.39, 3.09, 2.86, 1.98, 1.81
Quartz	S1–S11	17.8, 20.87, 24.3, 26.70, 36.06, 36.60, 39.50, 40.36, 42.51, 45.90, 50.20, 54.94, 55.38, 55.6, 60.0, 67.79, 68.00, 68.18, 68.37, 73.51, 75.70, 77.70	4.99, 4.26, 3.68, 3.34, 2.49, 2.45, 2.28, 2.23, 2.13, 1.98, 1.82, 1.67, 1.66, 1.65, 1.54, 1.38, 1.38, 1.377, 1.374, 1.29, 1.25, 1.23
Kaolinite	S1–S11	12.5, 17.80, 19.86, 23.13, 40.36, 45.90, 50.66, 55.7, 61.6, 64.06	7.15, 4.99, 4.49, 3.85, 2.23, 1.98, 1.80, 1.65, 1.50, 1.45
Montmorrilonite	S1, S2, S3, S4, S5, S6, and S11	19.8, 27.2, 35.03	4.49, 3.28, 2.56, 1.50
Crocidolite	not detected		
Chrysotile	S1–S11	12.1, 19.7, 24.3, 36.7, 60.1	7.30, 4.50, 3.65, 2.44, 1.53
Calcite	S1–S11	23.13, 29.41, 36.06, 39.50, 43.30, 47.50, 48.6	3.85, 3.07, 2.49, 2.28, 2.09, 1.91, 1.87
Vaterite	S1–S11	20.7, 25.2, 27.5, 32.2, 43.4	4.29, 3.54, 3.24, 2.78, 2.08
Dolomite	S1–S11	24.2, 31, 41.2, 49.5, 50.6, 51.2	3.68, 2.88, 2.19, 1.84, 1.80, 1.78
Hematite	S1–S11	19.9, 22, 24.3, 33.3, 35.7, 37.5	4.46, 4.04, 3.68, 2.69, 2.50, 2.34

**Figure 8:**
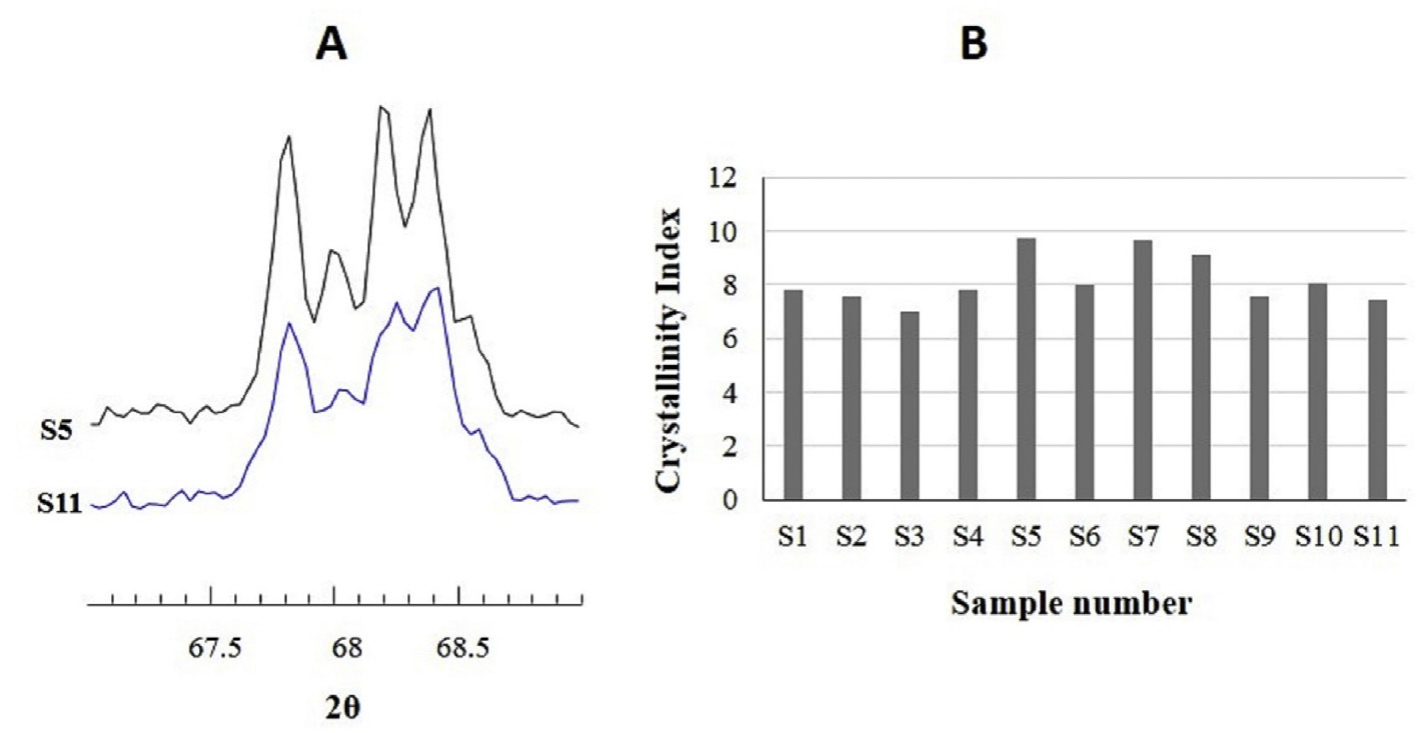
(A) XRD data for S5 and S11 plotted in the range of 2θ values of 67^o^ to 69^o^. (B) The calculated crystallinity index of all the samples.

Majority of intense peaks in XRD data represented quartz mineral, which is due to high crystallinity order of quartz. One of the approaches to measure the crystallinity of quartz is to measure the crystallinity index (CI) in the scale of 0–10; 0 referring to the amorphous material and 10 referring to the perfectly crystalline material [38]. The presence of five diffraction peaks in the range of 2θ value of 67^o^ to 69° indicated crystallized quartz [38]. The XRD pattern in the region of interest for samples S5 and S11 is shown in Figure 8A. We found quintuplet peaks indicating the presence of quartz of high crystalline order. The CI values of the dust samples ranged from 7 to 9.7 (Figure 8B). This indicated that in all samples studied silicate mineral of high degree of crystallinity was present.

### 4.4 Comparison of different data

The SEM-EDX data are consistent with XRD and FTIR data. For example, sample S4 consisted of minerals such as calcite, dolomite, vaterite, illite, montmorrilonite, kaolinite, chrysotile, hematite, and quartz. The types of elements required for the formula unit of these minerals are also found to exist in the EDX data (refer to Table 2 for sample S4). It is interesting to note that the single particle data for samples S2 and S3 (S2-1, S3-1, S3-2) contained different elemental composition than EDX spectrum collected for whole field of view (S2 and S3 in Table 2). The single particle spectrum S2-1 contained Ca, Si and Al as the metallic element indicating that the particle could be rich in calcite, vaterite, and quartz.

### 4.5 Source of minerals in dust

**Figure 9:**
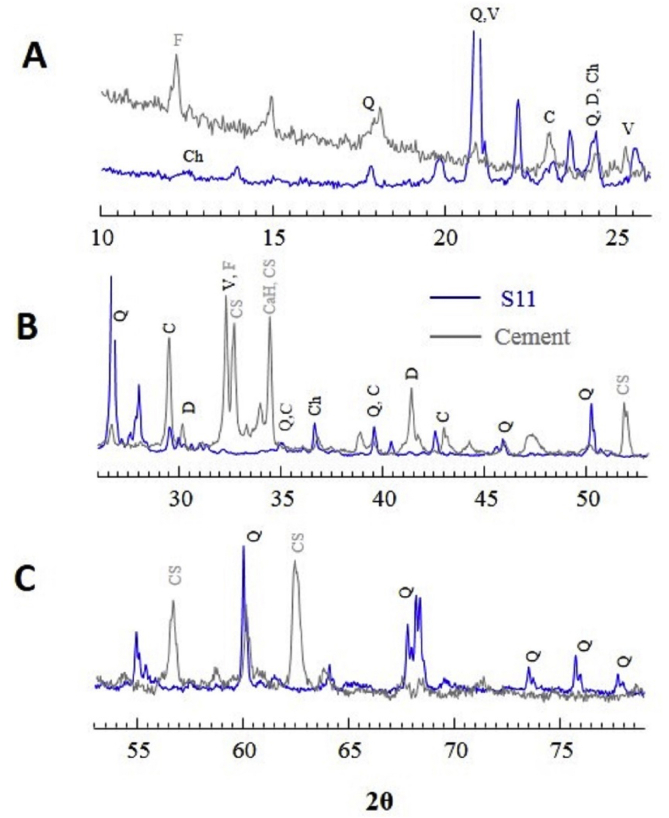
XRD pattern of cement sample (gray color) and sample 11 (black color). For clarity, the data are plotted in the range of 2θ value of 10°–26° in frame A, 26°–53° in B, and 53°–79° in frame C. For easy comparison the S11 peaks ~21° and 26.7° are truncated. Ch = chrysotile, Q = quartz, C = calcite, CS = calcium silicate, CaH = calcium hydroxide, D = dolomite, F = ferrite, and V = vaterite. The peaks that closely overlap in two samples are labeled in black color and the intense peaks present only in cement samples are labeled gray color.

In Kathmandu Valley, almost all structures are concretized. In this respect, cement can be one the sources of the minerals in the particulate matter. To explore this, we measured XRD data of three cement samples obtained from three major cement suppliers. The samples looked very similar in XRD and a representative datum along with sample 11 (S11) is plotted in Figure 9. For clarity, the data are plotted in the range of 2θ value of 10°–26° in frame A, 26°–53° in B, and 53°–79° in frame C. Interestingly, the cement samples also showed peaks characteristics of: i) crystalline silica (quartz), ii) carbonate minerals vaterite, calcite, and dolomite, iii) asbestiform mineral chrysotile. The cement samples also showed dominant peaks characteristics of calcium hydroxide, calcium silicate, and iron rich mineral ferrite (rich in hematite). These minerals are also reported in cement samples of other countries [70]. This observation indicates that cement could be one of the sources of quartz, vaterite, calcite, dolomite, and chrysotile minerals in the dust sample studied.

The earlier studies reported that the cardiovascular related diseases and deaths are increasing in Nepal [24, 71]. It is established fact that air pollution is one of the major contributors to respiratory and cardiovascular diseases [6, 72]. Our study showed that dust samples collected from the densely populated locations of Kathmandu Valley contain different minerals including crystalline silica (quartz) and asbestiform minerals chrysotile and crocidolite. Inhalation of crystalline silica and asbestos rich air cause different pulmonary and cardiovascular diseases and eventually to death. Without the information on concentration of minerals, exposure dose, and epidemiological studies, it is not possible to comment on the cardiovascular related health issues, however, we believe that the findings of this work will be important to understand the respiratory and cardiovascular related health issues in Kathmandu Valley.

Furthermore, we also like to mention the morphological and chemical analysis of particulate matter samples from different Asian cities other than Kathmandu. Study on the air samples collected from different cities of Saudi Arabia reported that particles having rod, irregular, and circular shape existed in the dust samples [73]. A study made in air dust samples of Agra, India showed the presence of mineral, soot, fly ash, tarballs, aluminosilicates/silica, fluorine, carbon rich, and Cl–Na rich particles [74]. A similar study in samples collected from rural and urban area of Delhi [75] and Lucknow [76] was also reported. Morphology and composition of particulate matter collected from Peshawar, Pakistan was made by Zeb et al [77]. In the study particles, based on morphology and composition, were classified as: aluminosilicates (23%), silica (12%), carbonaceous (49%), calcium rich (3%), chloride (2%), Fe/Ti oxides (3%), sulfate (5%), biogenic (3%). A long term study (2012–2014) on PM_2.5_ samples collected form twenty urban cities of China was reported by Liu et al [78]. It was found that the major PM_2.5_ constituents across all the urban sites were organic matter (26.0 %), elemental carbon (6.0 %), SO_4_^2−^ (17.7 %), NO_3_^−^ (9.8 %), NH_4_^+^(6.6 %), Cl^−^ (1.2 %), mineral dust (11.8 %), and unaccounted matter (20.7 %).

## 5 Conclusions

To summarize, we identified different minerals in dust samples collected from eleven core urban areas of Kathmandu Valley. We found carbonate minerals (calcite, vaterite, and dolomite) iron rich mineral (hematite), clay minerals (illite and kaolinite), crystalline silicate mineral (quartz), and asbestiform mineral present in all samples. The clay minerals montmorrilonite and asbestiform mineral crocidolite were found in seven and five samples, respectively. This observation was supported by EDX and XRD measurements. Electron microscopic measurements showed particles having very diverse morphology and very few particles of asbestiform type. The crystallinity index (CI) of quartz mineral in the samples ranged from 7 to 9.7 indicating that quartz of high crystallinity present in all the samples. The major elements in the dust samples were found to be C, O, Mg, Ca, and Si. The XRD data analysis in three different brands of cement samples showed the presence of crystalline silica (quartz), carbonate minerals (vaterite, calcite, and dolomite, and asbestiform mineral chrysotile; indicating that cement could be one of the sources of minerals in dust samples.

## Declarations

### Author contribution statement

Bhanu Neupane: Conceived and designed the experiments; Analyzed and interpreted the data; Wrote the paper.

Amita Sharma, Mahesh K. Joshi: Performed the experiments; Analyzed and interpreted the data.

Basant Giri: Analyzed and interpreted the data.

### Funding statement

This research did not receive any specific grant from funding agencies in the public, commercial, or not-for-profit sectors.

### Competing interest statement

The authors declare no conflict of interest.

### Additional information

No additional information is available for this paper.
